# The Influence of Hypoxia during Different Pregnancy Stages on Cardiac Collagen Accumulation in the Adult Offspring

**DOI:** 10.1155/2014/419805

**Published:** 2014-06-11

**Authors:** Lingxing Wang, Meimei Li, Ziyang Huang, Zhenhua Wang

**Affiliations:** ^1^Department of Neurology, Second Affiliated Hospital of Fujian Medical University, Quanzhou, Fujian 362000, China; ^2^Department of Cardiology, Second Affiliated Hospital of Fujian Medical University, Quanzhou, Fujian 362000, China

## Abstract

We evaluated whether the timing of maternal hypoxia during pregnancy influenced cardiac extracellular matrix accumulation in the adult offspring. Rats in different periods of pregnancy were assigned to maternal hypoxia or control groups. Maternal hypoxia from day 3 to 21 of pregnancy or day 9 to 21 of pregnancy increased collagen I and collagen III expression in the left ventricle of adult offspring (both *P* < 0.05). Maternal hypoxia from day 15 to 21 of pregnancy had no effect on adult collagen levels. Our results indicate that maternal hypoxia at critical windows of cardiovascular development can induce pathological cardiac remodeling in the adult rat offspring.

## 1. Introduction

Cardiovascular disease is a leading cause of death in developed countries [1]. Several risk factors, including smoking, hypertension, and high body mass index have been identified. These risk factors, however, are not sufficient to explain the prevalence of disease. Recently, the concept of fetal origins of adult disease has emerged as a possibility to explain the high incidence of cardiovascular disease. This concept hypothesizes that an adverse intrauterine environment during a critical window of fetal or infant development can exert long-term effects on adult tissue structure or function [2–4].

Maternal hypoxia during pregnancy in rats leads to changes in cardiac structure and function in adult male offspring [5]. Other animal models indicate that timing and severity of the fetal insult are critical to the programming of adult disease [6, 7]. In this report, we elucidate the influence of maternal hypoxia during different periods of pregnancy on cardiac collagen accumulation in adult offspring.

## 2. Methods

### 2.1. Animals and Hypoxic Protocol

All experimental procedures were in accordance with the National Institutes of Health guidelines and were approved by the Standing Committee on Ethics and Animal Experimentation at the Fujian Medical University (China). Virgin female Sprague-Dawley (SD) rats (250–280 g in body weight) were obtained from the Shanghai Experimental Animal Center, Shanghai, China. They were housed individually in standard rat cages under controlled temperature (22 ± 1°C) with lights on from 07.00 to 19.00 h. Food and water were provided* ad libitum*. The female rats were mated within the animal facility, and the vaginal smears were checked every morning. Pregnancy was confirmed by the presence of sperm-positive vaginal smears (day 0). The 24 pregnant rats were randomized to one of four groups: a hypoxia group from day 3 to day 21 of pregnancy (G3–21; *n* = 6), a hypoxia group from day 9 to day 21 of pregnancy (G9–21; *n* = 6), a hypoxia group from day 15 to day 21 of pregnancy (G15–21; *n* = 6), and a control group (G0; *n* = 6).

Maternal hypoxia was induced by the method described by Wang and colleagues [8]. Briefly, the pregnant rats assigned to the hypoxia groups were placed inside a plexiglass chamber (140 L) for 3 h per day during the light cycle. Nitrogen gas and compressed air were continuously infused into the chamber to maintain an oxygen concentration of 10 ± 1%. The oxygen concentration in the chamber was monitored using a portable gas analyzer that was calibrated daily (S-450; IST-AIM).

After birth, litters were reduced to 8 pups per dam to standardize the nutrient supply. Offspring were weaned at 3 weeks and housed in the animal facilities of Fujian Medical University (China). Experimental models have shown that male offspring are more susceptible to the adverse fetal environment [9, 10]; accordingly, only male offspring (2 or 3 rats per litter) were evaluated in this experiment.

### 2.2. Blood Pressure Measurements

The systolic blood pressure of the male offspring was determined 3 months (*n* = 12 in each group) and 5 months (*n* = 6 in each group) of age using the tail cuff method (RBP-1B, Clinical Medical Instrument Institute of Beijing Sino-Japan Friendship Hospital, Beijing, China). For the rats that were evaluated at 5 months of age, blood pressure was also measured at 3 months of age. The male offspring were trained for one week before the blood pressure measurements were taken. The average measurements from each animal on three separate days were taken to determine the individual mean blood pressure for that animal.

### 2.3. Tissue Collection

After being anesthetized with an intraperitoneal injection of pelltobarbitalum natricum (20 mg/kg), hearts were removed from male offspring at the age of 3 months (*n* = 6) and 5 months (*n* = 6). After determination of heart weight (HW), the left ventricle was carefully separated from the atria and the right ventricle and weighed. The wet weight of the left ventricle (LV) was recorded, normalized for body weight (BW), and expressed as the ventricular mass index. The LV tissue was divided into 2 sections. One section was fixed in 4% paraformaldehyde for histological analysis and the other section was snap-frozen in liquid nitrogen for protein analysis.

### 2.4. Histopathology

Tissues were dehydrated through graded alcohols and xylene and then embedded in paraffin. Sections were cut at 5 *μ*m thickness and stained with haematoxylin and eosin. The diameter of cardiomyocytes was determined by measuring the shortest transverse diameter of myocytes that were cut transversely with an intact membrane and a centrally located nucleus. The measurements were performed using ImageJ software.

### 2.5. Immunoblot Analyses

Immunoblotting was performed as described previously [5]. Briefly, LV tissue was homogenized in ice-cold lysis buffer. Crude homogenates were centrifuged for 5 min at 10000 g, and protein concentration of the supernatants was determined by the BCA method (Pierce Chemical Co). Supernatants were added to loading buffer and heat denatured by boiling for 3 min. Protein (25 ug) was electrophoresed in separate lanes on 8% SDS-PAGE. After electrophoresis, proteins were transferred onto nitrocellulose membranes and blocked in a 5% nonfat dried milk solution at room temperature. The blot was incubated with antibodies against collagen I or collagen III (1 : 100 dilution; Beijing Biosynthesis Biotechnology Company, China). A peroxidase-conjugated avidin secondary antibody was applied for visualization (1 : 5000 dilution; Santa Cruz Biotechnology, USA). The blots were stripped and reprobed with a *β*-actin antibody (1 : 2000; Beijing Biosynthesis Biotechnology Company, China) to normalize for protein loading. The integrative grayscale pixel area-density was captured with a CCD camera and quantified using Quantity One software.

### 2.6. Data Analysis

Data were expressed as mean ± SEM. A one-way ANOVA, followed by an SNK post hoc test, was performed to compare groups. A value of *P* < 0.05 was considered statistically significant.

## 3. Results

### 3.1. Systolic Blood Pressure of Male Offspring

At the age of 3 months and 5 months, we found significantly higher systolic blood pressure in male offspring from the G3–21 group compared with the G0 group (three months: G3–21 122 ± 3 mmHg versus G0 108 ± 3 mmHg, *P* < 0.05; five months: G3–21 129 ± 3 mmHg versus G0 115 ± 4 mmHg, *P* < 0.05). There were no significant differences in systolic blood pressure between the G9–21, G15–21, and G0 groups (three months: G9–21 116 ± 4 mmHg, G15–21 112 ± 3 mmHg, *n* = 12; five months: G9–21 117 ± 3 mmHg, G15–21 117 ± 3 mmHg; Table 1).

### 3.2. Body Weight and Left Ventricular Weight of Male Offspring

**Figure 1:**
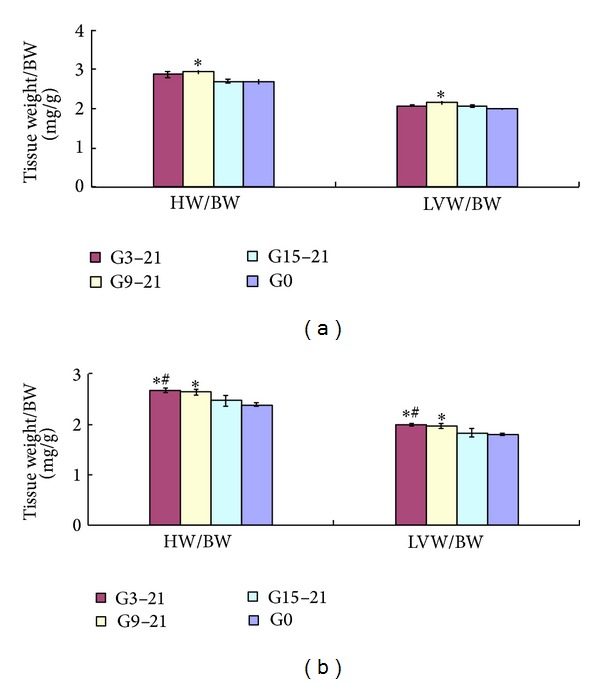
HW/BW ratios and LVW/BW ratios of rat male offspring at 3 months of age (a) and 5 months of age (b). All data are expressed as mean ± SEM, *n* = 6. HW, heart weight; BW, body weight; LVW, left ventricle weight. G3–21, maternal hypoxia group from day 3 to day 21 of pregnancy; G9–21, maternal hypoxia group from day 9 to day 21 of pregnancy; G15–21, maternal hypoxia group from day 15 to day 21 of pregnancy; G0, control group. *P < 0.05 compared to control group, ^#^ *P* < 0.05 compared to G15–21.

At 3 months of age, the HW/BW ratio and the LVW/BW ratio were significantly increased in the G9–21 male offspring compared with the G0 male offspring (HW/BW: G9–21 2.92 ± 0.06 versus control 2.67 ± 0.06, *P* < 0.05; LVW/BW: G9–21 2.15 ± 0.05 versus control 1.98 ± 0.02, *P* < 0.05, *n* = 6), while there was no significant difference between the G3–21, G15–21 and G0 groups (HW/BW: G3–21 2.86 ± 0.08, G15–21 2.69 ± 0.05, *n* = 6; LVW/BW: G3–21 2.07 ± 0.02, G15–21 2.06 ± 0.04, *n* = 6; Figure 1(a)). At 5 months, the HW/BW ratio and LVW/BW ratio remained higher in the G9–21 male offspring compared with G0 rats (HW/BW: G9–21 2.64 ± 0.06 versus control 2.39 ± 0.03, *P* < 0.05, *n* = 6; LVW/BW: G9–21 1.96 ± 0.05 versus control 1.80 ± 0.02, *P* < 0.05, *n* = 6). In addition, there was a significantly increased HW/BW ratio and LVW/BW ratio in male offspring from the G3–21 group compared with the G0 and G15–21 group (HW/BW: G3–21 2.68 ± 0.05 versus control 2.39 ± 0.03 or G15–21 2.47 ± 0.11, *P* < 0.05, *n* = 6; LVW/BW: G3–21 1.99 ± 0.03 versus control 1.80 ± 0.02 or G15–21 1.83 ± 0.08, *P* < 0.05, *n* = 6). The HW/BW ratio and the LVW/BW ratio in offspring from G15–21 group were not significantly higher compared with the G0 group (HW/BW: G15–21 2.47 ± 0.11, *n* = 6; LVW/BW: G15–21 1.83 ± 0.08, *n* = 6; Figure 1(b)).

### 3.3. Diameter of Cardiomyocytes from Male Offspring

**Figure 2:**
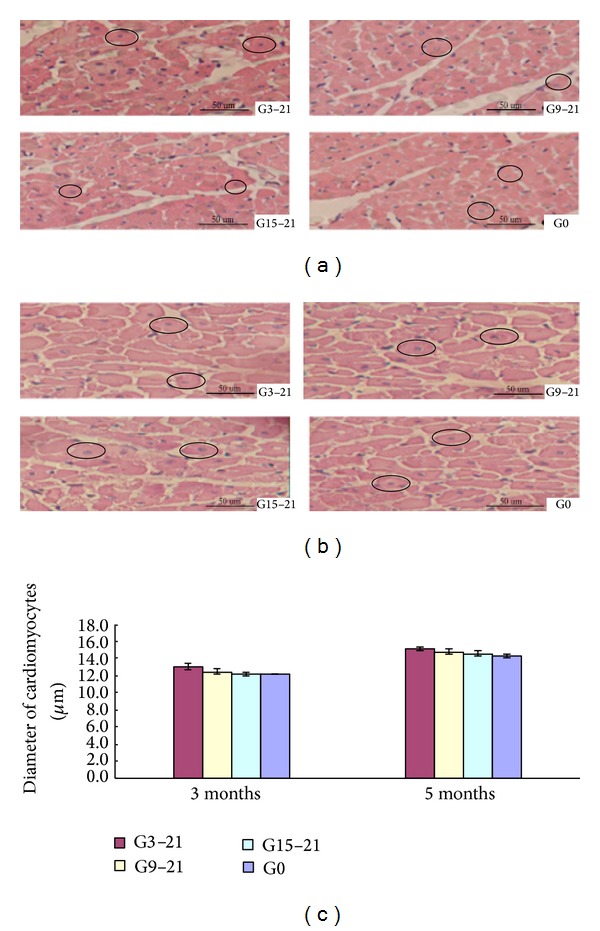
Representative light micrographs of cardiomyocytes stained with haematoxylin-eosin from male offspring at 3 months (a) and 5 months (b) of age. Examples of myocytes that met the criteria for evaluation are circled. Quantification of myocyte diameter from male offspring (c). All data are expressed as mean ± SEM, *n* = 6. No significant differences in the transverse diameter of cardiomyocytes from male offspring were seen among the groups. G3–21, maternal hypoxia group from days 3 to 21 of pregnancy; G9–21, maternal hypoxia group from days 9 to 21 of pregnancy; G15–21, maternal hypoxia group from days 15 to 21 of pregnancy; G0, control group. The circles in the picture indicate myocytes which met the criteria. Magnification for all photomicrographs is ×400.

The transverse diameter of the cardiomyocytes from the male offspring was not significantly different in the G3–21, G9–21, G15–21, and G0 groups at either 3 or 5 months (three months: G3–21 13.1 ± 0.4 *μ*m, G9–21 12.5 ± 0.3 *μ*m, G15–21 12.2 ± 0.3 *μ*m, G0 12.2 ± 0.1 *μ*m, *n* = 6; five months: G3–21 15.1 ± 0.2 *μ*m, G9–21 14.8 ± 0.3 *μ*m, G15–21 14.6 ± 0.3 *μ*m, G0 14.3 ± 0.2 *μ*m, *n* = 6; Figure 2).

### 3.4. Expression of Collagen I and Collagen III Protein

**Figure 3:**
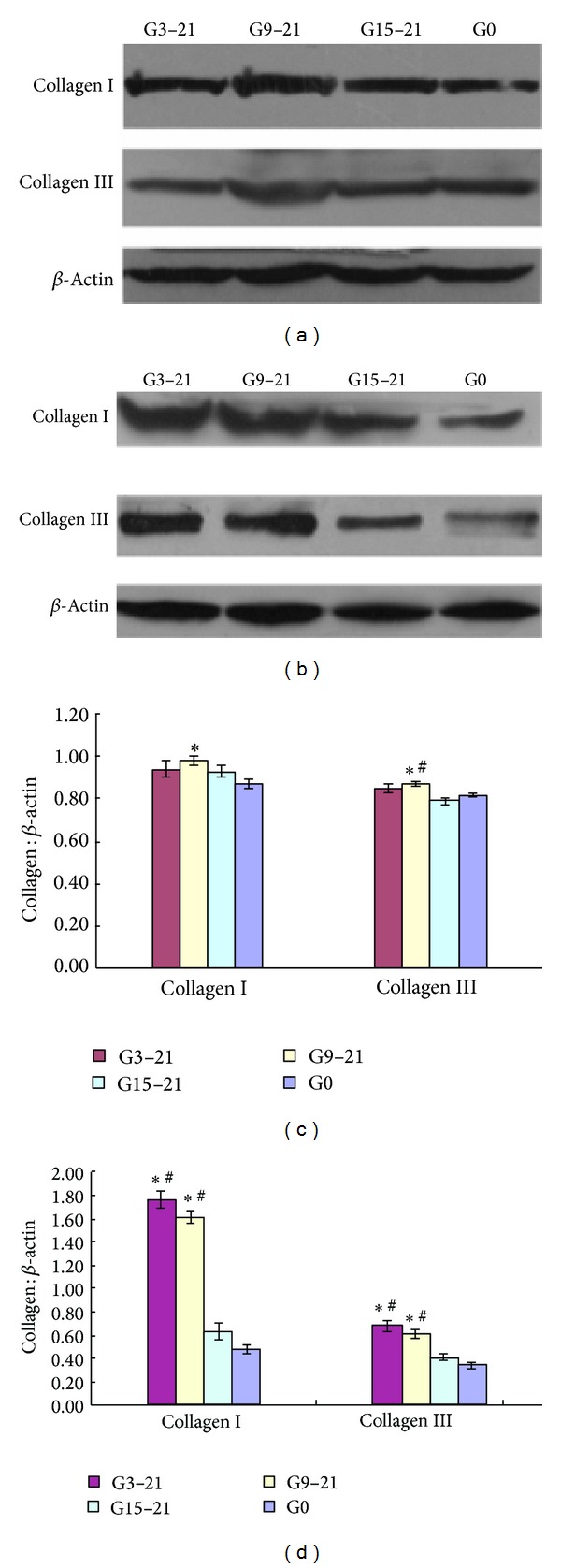
Representative immunoblots of collagen I and collagen III in the left ventricle of male offspring from the four experimental groups at 3 months (a) and 5 months (b) of age. Quantification of collagen I and collagen III is depicted for offspring at 3 (c) and 5 months of age (d). All data are expressed as mean ± SEM, *n* = 6. G3–21, maternal hypoxia group from days 3 to 21 of pregnancy; G9–21, maternal hypoxia group from days 9 to 21 of pregnancy; G15–21, maternal hypoxia group from days 15 to 21 of pregnancy; G0, control group. *P < 0.05 compared to control group and ^#^ *P* < 0.05 compared to the G15–21 group.

Collagen I protein expression was significantly greater in the LV tissue from the G9–21 male offspring at 3 months compared with the G0 male offspring (collagen I/*β*-actin: G9–21 0.98 ± 0.02 versus G0 0.87 ± 0.02, *P* < 0.05). The collagen III/*β*-actin ratio was also significantly higher in the G9–21 male offspring compared to either the G0 or G15–21 offspring (collagen III/*β*-actin: G9–21 0.87 ± 0.01 versus G0 0.82 ± 0.01 or G15–21 0.79 ± 0.02, *P* < 0.05, *n* = 6). There was no significant difference in the expression of collagen I and collagen III between the G3–21, G15–21, and G0 groups (collagen I/*β*-actin: G3–21 0.94 ± 0.04, G15–21 0.93 ± 0.03; collagen III/*β*-actin: G3–21 0.85 ± 0.02, *n* = 6; Figures 3(a) and 3(c)).

In the 5-month-old offspring, expression of collagen I and collagen III was significantly increased in the LV tissue from both the G3–21 and G9–21 male offspring compared with the G0 or G15–21 male offspring (collagen I/*β*-actin: G3–21 1.76 ± 0.07 or G9–21 1.61 ± 0.05 versus G0 0.48 ± 0.04 or G15–21 0.63 ± 0.07, *P* < 0.05, *n* = 6; collagen III/*β*-actin: G3–21 0.68 ± 0.05 or G9–21 0.61 ± 0.04 versus G0 0.34 ± 0.03 or G15–21 0.41 ± 0.03, *P* < 0.05, *n* = 6), but there was no significant difference in collagen expression between the G15–21 and G0 groups (Figures 3(b) and 3(d)).

## 4. Discussion

Our previous studies have shown that maternal hypoxia with an oxygen concentration of 10 ± 1% induces significant hypoxemia without CO_2_ retention and acidosis, and a hypoxic duration of 3 h per day reduces neonatal size and organ weight but does not reduce maternal food intake [8, 11]. Our present study demonstrated that maternal hypoxia during different periods of pregnancy differentially altered collagen accumulation in the adult male offspring.

The LVW/BW ratio is an established index of cardiac hypertrophy [12]. Our study showed that maternal hypoxia from days 3 to 21 and from days 9 to 21 of pregnancy, but not from days 15 to 21, resulted in cardiac hypertrophy in the male offspring. Pathologic cardiac remodeling might be characterized by the abnormal accumulation of collagen [13]. In our study, there was no difference in the diameter of cardiomyocytes from the male offspring, and the expression of both collagen I and III dramatically increased in the hypertrophic ventricle. This suggests that the accumulation of collagen may be the primary mechanism for the increased wall thickness. One limitation of this study was that only one index of cardiac hypertrophy was measured, namely, the HW or LV mass to BW ratios. Additional markers of hypertrophy would have confirmed this result.

Not only does cardiac collagen provide a structural framework for cardiomyocytes and the coronary blood vessels, but also collagen determines the physiological performance by modifying myocardial stiffness and tolerance to deformation [14]. The cardiac collagen matrix remodels with age or pathology [15]. This remodeling often includes changes in the quality and quantity of the collagen matrix. The adverse accumulation of collagen fibers will increase cardiac stiffness and affect diastolic function. With further deposition of collagen, the systolic motion, coronary flow reserve, and electrical activity might then be affected and contribute to pathology [16, 17].

Collagen I and collagen III are the major collagen fibers in adult hearts, and pathological remodeling is associated with increased collagen I and collagen III. For these reasons, we focused on detecting the two types of collagen and did not detect cross-linked versus noncross-linked collagen. Our study demonstrated that collagen I and collagen III were increased in the hearts exposed to maternal hypoxia started in early or midpregnancy times, compared with the control group. Studies have suggested that prenatal deficiencies of metabolic substrates including oxygen or nutrients will affect postnatal cardiac structure. In our study, maternal hypoxia might have led to abnormal regulation of cardiac collagen by directly influencing metabolism [5, 18]. Moreover, previous studies have shown that the rat cardiovascular system differentiates and develops most from the 9th to 13th embryonic day and that this time period is regarded as a critical window for cardiovascular development in rat embryos [19, 20]. It is interesting to speculate that the changes of internal environment around the critical window of cardiovascular development will have the most influence on the cardiac structure of the rat offspring. That would explain why there was no increase of collagen deposits in the hearts of the G15–21 hypoxia group that was exposed outside the critical development window, from day 15 to day 21. Further studies are necessary to test this hypothesis.

Abnormal collagen accumulation is also associated with hypertensive heart disease, but in the G9–21 maternal hypoxia group there was collagen deposition in the hearts of offspring in the absence of hypertension. This result indicates that collagen deposition caused by maternal hypoxia might be independent of hypertension, which is consistent with previous studies [5, 21].

## 5. Conclusions 

Maternal hypoxia around a critical window of cardiovascular development might have the most important influence on cardiac collagen deposition of the adult rat offspring. Pathology occurs without cardiomyocytes hypertrophy and it appears to be independent of hypertension.

## Figures and Tables

**Table 1:** Systolic blood pressure of male offspring.

Group	3 months (*n* = 12)	5 months (*n* = 6)
G3–21	122 ± 3*	129 ± 3*
G9–21	116 ± 4	117 ± 3
G15–21	112 ± 3	117 ± 3
G0	108 ± 3	115 ± 4

Values are expressed as mean ± SEM mm Hg, *P < 0.05 compared with the control group. At 3 months of age, *n* = 12 for each group; at 5 months of age, *n* = 6 for each group.

## Abbreviations

LV: Left ventricle
LVW: Left ventricular weight
BW: Body weight
HW: Heart weight.
